# Outbreaks of Avian Influenza A (H5N2), (H5N8), and (H5N1) Among Birds — United States, December 2014–January 2015

**Published:** 2015-02-06

**Authors:** Michael A. Jhung, Deborah I. Nelson

**Affiliations:** 1 Influenza Division, National Center for Immunization and Respiratory Disease, CDC; 2 Animal and Plant Health Inspection Service, U.S. Department of Agriculture, Fort Collins, Colorado

During December 15, 2014–January 16, 2015, the U.S. Department of Agriculture received 14 reports of birds infected with Asian-origin, highly pathogenic* avian influenza A (HPAI) (H5N2), (H5N8), and (H5N1)† viruses. These reports§ represent the first reported infections with these viruses in U.S. wild or domestic birds. Although these viruses are not known to have caused disease in humans, their appearance in North America might increase the likelihood of human infection in the United States. Human infection with other avian influenza viruses, such as HPAI (H5N1) and (H5N6) viruses and (H7N9) virus, has been associated with severe, sometimes fatal, disease (1–3), usually following contact with poultry.

The 14 HPAI H5 detections, seven (H5N2), six (H5N8), and one (H5N1), occurred in five northwestern states (California, Idaho, Oregon, Utah, and Washington). Outbreaks occurred in five domestic, backyard flocks, two captive wild birds, and seven wild aquatic birds. All backyard flocks were destroyed after identification of HPAI H5 virus. Of 24 persons reporting exposure to infected birds, one person developed influenza-like illness (ILI) after exposure but subsequently tested negative for influenza.

CDC has developed testing (http://www.cdc.gov/flu/avianflu/severe-potential.htm) and influenza antiviral prophylaxis (http://www.cdc.gov/flu/avianflu/guidance-exposed-persons.htm) guidance for persons exposed to birds possibly infected with HPAI H5 viruses. Until more is known about these viruses, CDC is taking a cautious approach, and recommendations are largely consistent with guidance for influenza viruses associated with severe disease in humans. Clinicians and public health workers should consider the possibility of infection with HPAI H5 viruses in patients with ILI who have had recent contact with sick or dead birds, especially in areas where these viruses have been identified. Persons exposed to birds infected with HPAI H5 should be monitored for ILI for 10 days after their last exposure, and influenza antiviral prophylaxis may be considered to prevent infection. Persons who develop ILI after exposure to HPAI H5-infected birds should be tested immediately for influenza by the state health department. State health departments are encouraged to investigate all possible human infections with HPAI H5 virus and should notify CDC promptly when testing for influenza in persons with ILI who have been exposed to birds possibly infected with these viruses.

## Footnotes

* Highly pathogenic refers to the spectrum of illness seen in birds.

† The H5N1 virus isolated from a U.S. wild bird is a new mixed-origin virus (a reassortant) that is genetically different from the avian H5N1 viruses that have caused human infections with high mortality in several other countries (notably in Asia and Africa). No human infections with this new reassortant H5N1 virus have been reported.

§ Available at http://www.aphis.usda.gov/wps/portal/?urile=wcm:path:/aphis_content_library/sa_our_focus/sa_animal_health/sa_animal_disease_information/sa_avian_health.
